# Automatic position monitoring of endotracheal breathing tubes using a magnetic sensor array

**DOI:** 10.1186/s12938-025-01441-1

**Published:** 2025-09-15

**Authors:** Till Riemschneider, Thorben Schüthe, Robert Werdehausen, Thomas Schilling, Thomas Hachenberg

**Affiliations:** 1 Department of Anesthesiology and Intensive Care Medicine, Medical Faculty, Otto von Guericke University of Magdeburg, Leipziger Str. 44, 39120 Magdeburg, Germany; 2 Faculty of Engineering and Computer Sciences, University of Applied Sciences Hamburg, Berliner Tor 7, 20099 Hamburg, Germany; 3 Center of Anesthesiology and Intensive Care Medicine, Klinikum Magdeburg, Birkenallee 34, 39130 Magdeburg, Germany

**Keywords:** Endotracheal Tubus, Long-Term Ventilation, Intensive Care Monitoring, Tubus Dislocation, Undesired Extubation, Magnetic Localization, Magnetic Tracking, Magnetic Sensor Array

## Abstract

**Background:**

Dislocation of an endotracheal tube (ETT) during invasive ventilation can lead to serious events such as unilateral ventilation or unintentional extubation. The correct position of the endotracheal tube is determined visually. X-ray imaging or invasive procedures such as bronchoscopy are established for repeated position verification. However, these measures are time-consuming and only provide a limited number of snapshots. A new monitoring method can recognize dislocations of the ETT. The proposed system operates automatically without the need for continuous staff awareness or interaction.

**Materials and methods:**

A ring-shaped permanent magnet is attached to the ETT. A small device is placed extracorporeally on the patient to detect the magnetic field. This device uses 64 magnetic sensors arranged as a sensor array in an 8x8 matrix. The sensor signals are digitally converted, enabling the position of the ETT with the attached magnet to be determined by software. Two processing methods (image similarity and localization) are tested for monitoring. The prototype system detects displacements with millimeter scale positioning deviations in our tests.

**Results:**

Our system triggers an alarm upon detecting an impermissible dislocation, complete extubation, or unintended bronchial intubation. The proposed methods were validated on a sensor array prototype and assessed through a dedicated experimental setup. The results are promising and could lead to further development towards clinical usability.

**Conclusion:**

Early warnings would be particularly advantageous, even for minor or beginning dislocations of the ETT. An automated continuous tube monitoring process could help reduce the workload of the staff and improve patient safety.

## Introduction

**Fig. 1:**
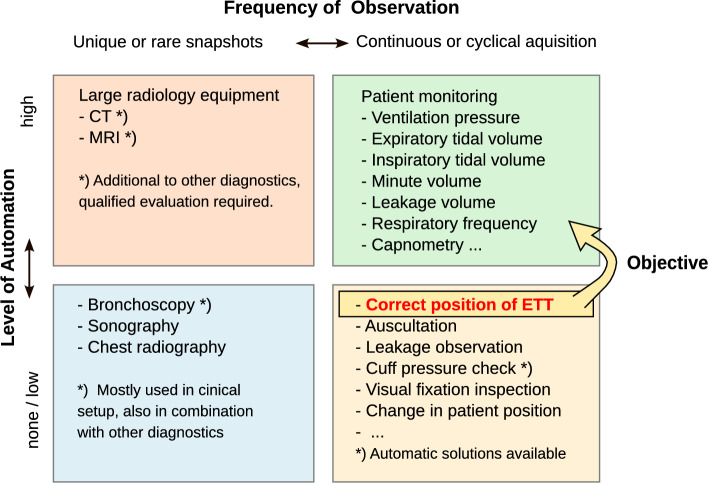
Categories for ETT placement testing and patient monitoring: The proposed approach is intended to complement the continuous monitoring methods and allow fully automated operation

**Fig. 2:**
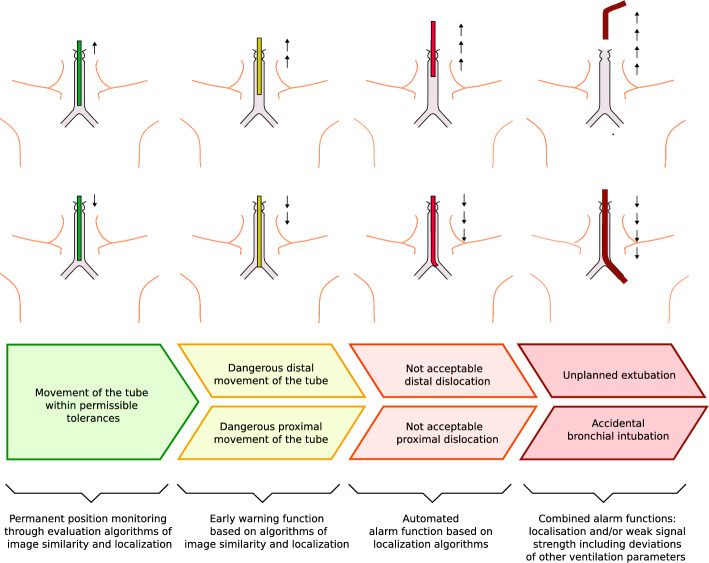
Stages of warning and alarm levels for ETT dislocation

Dislocation of an endotracheal tube can lead to serious events such as unilateral ventilation or unintentional extubation. The initial positioning and correct placement of the endotracheal tube (ETT) can be determined using various methods. The gold standard is laryngoscopic visualization of regular intubation [1–3]. Additional tools to prove intubation include abdominal auscultation, ultrasound procedures [4], capnometry [5], transthoracic impedance procedures [6] and chest radiographs [7, 8] as well as bronchoscopy [9]. It is common practice to check the tube insertion depth according to visible markings on the tube. The markings can be read in relation to defined anatomical landmarks (usually rows of teeth or corners of the mouth) during physical examination or auscultation. These visual inspections are carried out at regular intervals or in the event of a ventilator alarm. This ensures that the tip of the ETT remains optimally positioned at all times, typically 2 –6 cm above the carina. A position that is too high placed risks of tube cuff herniation, airway leakage and unwanted extubation. Positioning it too low increases the risk of bronchial intubation and one-sided ventilation and atelectasis [10].

Reliance on visual inspection places a substantial cognitive burden on clinical staff and may be impractical, particularly during head and neck interventions in which surgical drapes obstruct direct observation. Moreover, timely detection and accurate classification of complications at the bedside necessitate advanced training and clinical experience, thereby increasing the requirement for highly qualified personnel.

Currently available automatically recognized alarm events (such as loss of capnometry) provide only non-specific indications of tube position and are also time-delayed in relation to the root cause.

Therefore, a system for non-invasive and continuous monitoring of the tube position is desirable for anesthesia procedures and critical care medicine. The vision is a fully automatic and easy-to-use system that operates continuously with minimal need for personnel intervention, see also Fig. 1.

Recently, there has been a particular interest in re-examining this issue. Chhina et al. [11] demonstrated that unplanned extubations and necessary re-intubation could be observed approximately three times more frequently when ventilating SARS-CoV-2 patients. This was partly due to the challenge of sedation management for patients and partly due to stressful situations for the staff. Regarding undesired extubation events, Berkow et al. [12] highlight the particular danger these pose to medical staff due to the high risk of self-infection. They noted that the introduction of additional technical monitoring methods could enable faster responses and help prevent such events.

Likewise, Fig. 2 illustrates distinct phenomena associated with tube displacement and outlines their classification into warning and alarm levels. The objective of the early experimental study presented in this paper is to develop a dedicated monitoring approach capable of detecting tube dislocations in either direction and of any clinically relevant magnitude without delay, including at early stages.

**Fig. 3:**
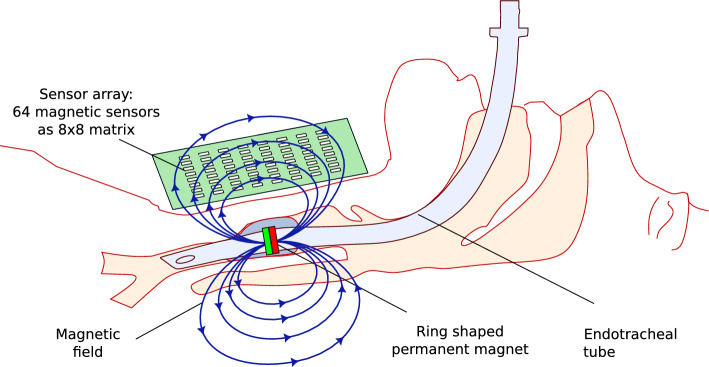
Concept of the proposed monitoring system—continuously operating localization of a permanent ring-magnet placed at the ETT

## Results

### Proposed concept

This article presents a concept and a functional demonstration for a compact magnetic field detection device containing an array of magnetic sensor chips. The field source is a small ring-shaped permanent magnet attached to the endotracheal tube. The static magnetic field passes unhindered through biological tissues without interaction. A magnetic sensor array is positioned externally in a suitable extracorporeal location. It continuously captures the magnetic field generated by the permanent magnet, and the acquired data are used to calculate the current position of the endotracheal tube. The integrated signal processing system automatically triggers a warning or alarm in the event of tube dislocation. Fig. 3 outlines the basic principle.

The possible pathway of the tube follows the anatomy of the trachea. Any movement may occur in two distinct directions, either proximally (towards the insertion site) or distally (with increased insertion depth). The recognition and indication of the appropriate reaction to these stages of dislocation defines the tasks a proposed monitoring device should fulfill, see also Fig. 1. Exceeding defined threshold values for the tube movement triggers these functions.

### Magnetic localization

Magnetic tracking and localization systems have been established for numerous medical applications, with commercial systems available for diagnostics or interventions. Most of these tracking systems utilize alternating electromagnetic fields and extracorporal field generators [13, 14, 16]. However, their complexity and cost make them unsuitable for continuous tube position monitoring during long-term ventilation on conventional ICU beds.

Although the use of permanent magnets as static field sources is still experimental, studies by Sun et al. [17] and Wichakool et al. [18] explored their use in assisting the initial placement of gastric tubes, ETTs, and catheters. In contrast, our system is designed for continuous and fully automated monitoring during long-term clinical use. A strong permanent magnet is required to ensure reliable sensor detection. From a physical perspective, this implies a large magnetic moment, which is directly related to the size or mass of the magnet. However, the anatomical dimensions of the trachea and the endotracheal tube impose clear constraints [19]. To account for this, only small magnets were used in our experimental investigations. By using neodymium as the magnet material, a high magnetic field strength can be achieved despite the compact dimensions. The use of a ring or hollow cylinder shape preserves the tube’s internal lumen. Therefore, the integration of the magnet ring is not expected to impair gas exchange or the functional passage of the tube. An axial direction of magnetization is advantageous for rotational symmetry. The magnet was mounted at the distal end of the tube. The cuff region proved to be a favorable location for placement.

**Fig. 4:**
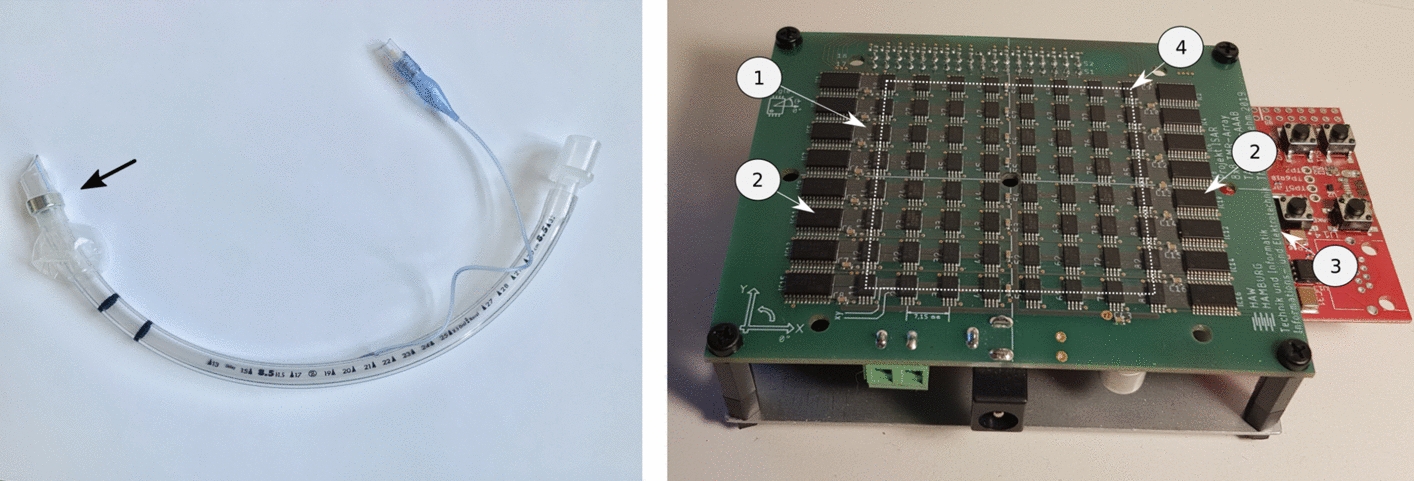
Key components of the monitoring system. Right: Prototype of the sensor array [21, 33]. (1) 64 magnetic sensors [20] (2) Multiplexer chips for the signals of the sensors (3) Microcontroller for control and digitization (4) Sensor matrix 50 $\times$ 50 mm$^2$ Left: Endotrachial tube with ring-magnet

**Fig. 5:**
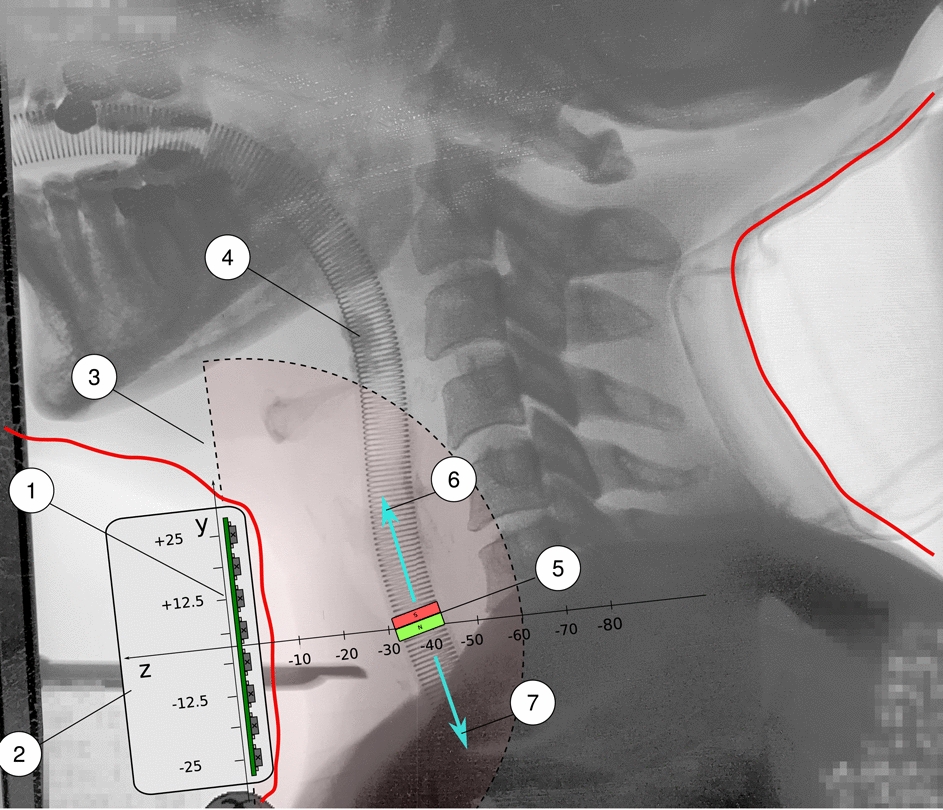
Placement of the sensor array on the patient (1) Sensor array seen from the side, (2) Monitoring device containing the sensor array, (3) Marking of the detection area, (4) Woodbrigde tube, (5) Preferred position of the magnet at the ETT near or inside the cuff, (6) Distal movement direction of ETT, (7) Proximal movement direction of ETT

A sensor array comprising 64 magnetic sensors was developed in an 8$\times$8 matrix configuration on a circuit board. This prototype represents a significant increase in the number of sensors compared with those employed in previous tracking systems. Moreover, these modern sensor chips [20] , which are based on the tunnel magnetoresistance effect (TMR) effect, exhibit comparatively high sensitivity. However, the sensor chips are available as low-cost mass products, as is the integrated microcontroller, see Fig. 4. This means that the technical effort remains reasonable while still providing extensive measurement data of the magnetic field. A schematic illustration of the interaction between the magnet on the tube and the sensor array positioned on the patient is shown in Fig. 5.

For this purpose, fixation prototypes were manufactured using additive manufacturing techniques with 3D-Printers and evaluated with intubation training phantoms. The localization procedure and the optional visualization takes place on a connected personal or laptop computer. Further technical details on the electronics and the measurement principle can be found at Schüthe [21, 22].

Two signal processing methods are presented for determining of the discrepancies between the current measured values of the sensor array and the initially stored reference data set. Two different algorithmic paradigms are evaluated: one based on image similarity metrics and the other on spatial localization calculations in the magnetic field. These approaches are not only contrasted conceptually, but also evaluated through implementation in dedicated software frameworks and tested under a well-defined experimental setup. This approach aims to demonstrate their practical limits of applicability, residual positioning errors, and achievable operational speeds under controlled conditions.

The prototype system provides a robust proof of principle, demonstrating that both the required localization accuracy and detection range for the monitoring tasks can be achieved.

## Conclusion

The proposed concept is currently under evaluation with a focus on clinical perspective [23], accompanied by the identification of relevant technical and practical refinement steps.

ETT displacement, particularly accidental extubation, is a severe problem in clinical anesthesia and critical care medicine. The automation of position continuous monitoring may improve patient care and reduce staff workload, because a major part of the real stress in ICU consists of the seamlessly required awareness. This effect is hard to quantify and need statistical observations, for our system there are no clinical data available up to now.

Overall, the proposed system can be seen as a dedicated addition to general monitoring in anesthesia and ICU environments, probably with a certain gain in time for functional ventilation alarms. As already illustrated in Fig. 1, an integration into the fully automated methods is being pursued, without placing any additional continuous demands on staff attention.

One notable advantage lies in the use of an exceptionally high number of 64 magnetic chip sensors. The larger sensor array – together with the proposed localization procedure – allows for precise tracking of the ETT relative to its initial reference position. The required computing time was very low and not limiting for the control task, while the costs for the hardware components can be kept low. Dimensions of the sensor array prototype permit the fabrication of a compact, extracorporeal detection device for direct attachment to the patient. Tests of the prototype and methods implemented in the software demonstrated the general functionality of the approach, which can serve as a basis for further technical improvements.

The DC field of the permanent magnet does not induce any eddy currents on the surrounding tissue and biological material. Therefore, there is no harmful exposure even during continuous operation in long-term use. This is in contrast to regularly repeated X-ray exposures.

The overall effort might be justified with regard to the preventable disadvantages of a prolonged ventilation period and possible additional complications such as nosocomial pneumonia or undesired extubation [24].

When the possibility of early warning of incipient tubal dislocation is confirmed in clinical practice, this should lead to a relevant reduction in currently occurring undesired extubation. In particular, the potential of delivering early warnings with low thresholds of movement appears to be very valuable, as early countermeasures may limit the need for complete tube re-positioning.

If the proposed monitoring system proves to be clinically effective, a positive effect on the complication rate and workload in the ICU setting can be expected. The prospect of a positive contribution to intensive care patient care should motivate in-depth research beyond the early experimental stage presented here.

## Methods

### Magnets and sensor array

Commercially available permanent magnets were chosen for mechanical compatibility with the tube. We choose ring-shaped neodymium magnets with large central lumen, ensuring there is no or negligible interference with the airflow.

Their magnetic moment is sufficient to act as a reliable field source, ensuring robust sensor signal detection. Furthermore, we selected axially magnetized magnets and embedded them in a fixed orientation in the tube materials. The deployment environment was considered when coating the neodymium magnets in medical-grade polymer.

In the experiments, two hollow cylindrical neodymium magnets (grade N52) with masses of 2.0 g and 5.6 g were used, corresponding to outer diameters of 9 mm and 15 mm, respectively. The inner diameters are 6.6 mm and 9 mm.

An unusually large number of 64 magnetic sensors is employed within the sensor array, setting it apart from known configurations in the literature.

The high count of sensors produce a densely sampled magnetic field, with the collected data forming a spatially resolved vector matrix. The matrix corresponds to the physical positions of the sensor chips in the plane of the printed circuit board.

In addition to the manifold of sensor chips in the array, a remarkably difference to established magnetic tracking systems is the permanent magnet as source a DC-field. One objective of our experiments was to prove this much simpler configuration wether it can fulfill the requirements of the monitoring task.

Moreover, the applied modern sensor chips type TAS2141/2143 are produced by TDK [20], which are based on the tunnel magnetoresistance effect (TMR) effect. They exhibit comparatively high sensitivity in two geometrical axis. As a result, the signal strength is sufficient to allow direct routing through electronic switches (analog multiplexer, standard type 74HC4067) to the analog-to-digital converter, eliminating the need for additional amplification. Switching control and data acquisition are handled by a modern microcontroller with integrated analog-to-digital converters. For our experiments we use the TM4C1294 from Texas Instruments as part of an evaluation board named Tiva C Series Connected LaunchPad EK-TM4C1294XL. It transmits the digitized values to a personal computer via an USB interface. On this computer, the subsequent signal processing steps are performed with dedicated software.

However, the sensor chips are available as low-cost mass products, as is the integrated microcontroller, see Fig. 4. This means that the technical effort remains reasonable while still providing extensive measurement data of the magnetic field.

### Sensor data processing

Initially Stored Reference Data: Monitoring should begin only after successful intubation and confirmation of correct tube placement. A prior verification must indicate reliable signs of proper intubation.

This initial position then serves as the reference location for the automatic monitoring system. It is first communicated to the system as a distinct reference point, for which an initial data set is stored.

A dislocation is detected by the monitoring system based on deviations between the current measurement data and the stored reference data set. If these deviations exceed predefined threshold values, a warning or alarm is triggered. If the deviations remain within a tolerable range, the tube position is considered correct and no alarm is issued.

Accurate acquisition of the reference position can be supported through vector arrows or color maps visualizations combined with an indication of sensor signal strength. Further details and few representative examples are provided later in Fig. 9.

**Fig. 6:**
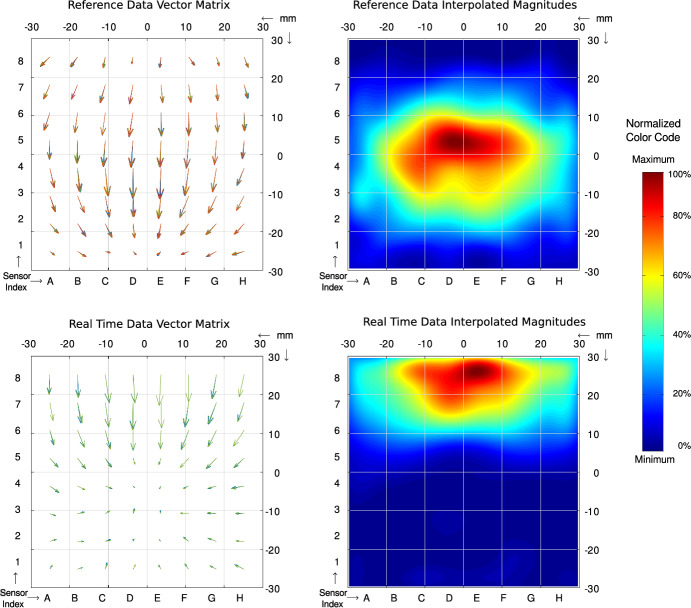
Visualization and demonstration of image similarity procedure. The vectors in the matrix indicate the direction and the strength of the magnetic field detected by each sensor. The data are also multiple interpolated and color coded. The shown result data are given in arbitrary units; they are scaled and normalized for best visualization. Top: Initial reference in central position Bottom: Current sensor values in a dislocation position of approx. 25–28 mm

Image Similarity Procedure: The measurement data from the sensor array can be analyzed in two dimensions. Direct vector field representations of sensor values using arrow matrices are hard to interpret visually. For this purpose, the vector matrix of the measured values is reduced to a matrix scalar values. Each scalar value can be the magnitude of the total vector or the magnitude of only one vector component. These matrices are displayed as false color images. Fig. 6 shows an example of recorded initial reference data and live real time data, acquired with our prototype sensor array.

Multiple interpolation steps insert intermediate values, resulting in images with a resolution of 113$\times$113 pixels or 225$\times$225 pixels. This higher resolution makes the images easy for a human to perceive and is suitable for visual comparison with each other. Color-coded and interpolated maps allow for intuitive localization of the magnet, with areas of peak sensor output values forming a distinct “cloud” in the image.

However, only positions within the limits of the dimensions of the sensor array are recognizable. If the displacement is only slight, this and the direction of displacement can be easily estimated via visual comparison with a reference image.

The visualization is helpful for the initial recording of a correct reference position, but is not required for the automatic monitoring function.

In this mode, a calculated similarity measure of the current image and the reference image serves as an indicator for the dislocation distance. The automatic function is based on the ongoing determination of this calculation. While the definition of image similarity is generally considered a non-trivial problem, it can be addressed with relative ease in the case of visualized magnetic fields. The similarity measure can be reduced to a simple numerical value. In the tests, the empirical correlation coefficient of the absolute values of the data matrices were well suited as indicator.

For this, each new aquired data frame is rendered and compared with the stored reference data frame using the cross-correlation coefficient. To be independent from the absolute magnitudes we choose the normalization as known as Pearson correlation coefficient.

The coefficient is significantly dependent on the movement of the field source, corresponding to the dislocation of the tube. Because this scalar metric drops sharply even for millimeter-scale shifts, simple thresholding of the coefficient yields fast early-warning and alarm triggers without needing full 3-D localisation.

The similarity determination can also be supported by separate correlation coefficients of the vector components in the x-axis and the y-axis of the sensor matrix.

This comparison of the measured values with the reference image requires negligible computing time. The calculation can thus be performed multiple times per second, enabling real-time monitoring.

If the correlation coefficient between the reference image and current measured image falls below predefined threshold values, an early warning or alarm will be triggered to indicate tube dislocation.

**Fig. 7:**
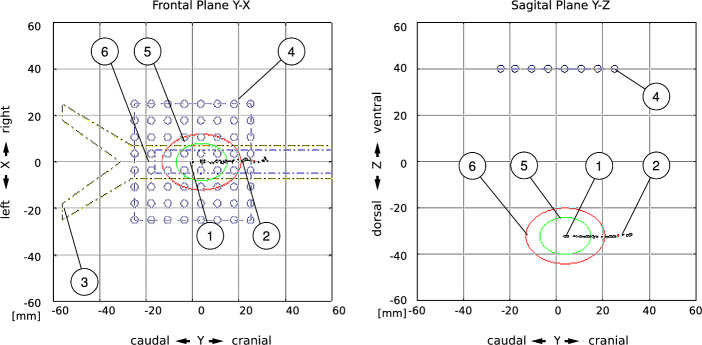
Proposal for software visualization of the localization tracking. The diagram in two planes of the patient coordinate system. The trachea, the tubus and the sensors are drawn schematically. (1) Initial reference position of the magnet, (2) Track of the magnet movement during a slow dislocation of ETT, (3) Simplified trachea outline, (4) Positions of the 64 sensor chips, (5) Warning threshold for dangerous dislocations, (6) Alarm threshold for not acceptable dislocations

Localization Procedure: A further procedure was investigated which analyses the sensor array data in three dimensions. For this, a complete localization determination is carried out. The spatial position and orientation of the permanent magnet in relation to the sensor array are calculated continuously.

In magnetic localization, calculation methods utilize a comparison of the current sensor data with a model of the permanent magnet. The model assumes that the permanent magnet is an ideal magnetic dipole. This dipole model concept is a point source without spatial extension, but with a direction and strength of magnetization in space. It is described by the magnetic moment as a vector.

The calculation of field strength is possible in the case of known location relationships and magnetization parameters through the application of the dipole equation [25]. This is known as the solution to the forward problem. The calculation in the opposite direction is not a straightforward process. This direction corresponds to the conclusion from the measured values to the position and orientation of the dipole. This task is referred to as the inverse problem. It is possible to apply a numerical optimization procedure, whereby a cost function is minimized. The cost function is also known as an error function or a distance norm. This optimization task can be solved numerically via an iterative procedure. Details of iterative methods for locating objects using magnetic tracking and navigation systems are discussed in the literature [26–31].

Our system models the ring magnet as an ideal magnetic dipole and continuously matches calculated field values to the 64 sensor readings, so all six pose parameters (x, y, z, plus three orientation angles) are estimated in real time. An optimizer algorithm perturbs these parameters step-wise and accepts only moves that shrink the residual error, converging on the magnet’s true 3-D position with sub-millimeter accuracy.

**Fig. 8:**
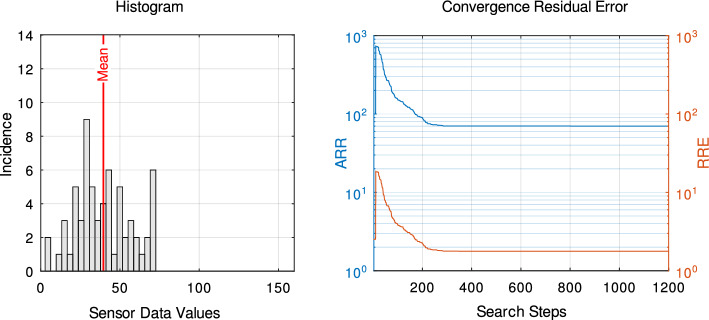
Example diagrams for localization procedure results - Left: Histogram of measured sensor values including all sensor element of the array, red dotted line mean of sensor values - Right: Convergence of the residual error between dipole model and measured values for all steps in the optimization algorithm, blue: absolute residual error (ARE) orange: relative residual error (RRE) normalized with the mean of sensor data values

Direct gradient descent, while being the most straightforward approach, suffers from the statistic nature of the measurement data. We illustrate this in Fig. 8 using a histogram. A broad distribution is beneficial in this context. In contrast, a narrow distribution with a low mean typically indicates a distant or complete removed magnetic source. This happens in the case of complete extubation. Convergence must be evaluated in relation to the overall signal. Therefore, we present the convergence curves of both the absolute residual error (ARE) and the relative residual error (RRE), the latter being a more significant indicator of convergence quality.

Based on comparative testing of alternative approaches, the developed software relies on stochastic “random walk” for optimization. This approach makes very small, random adjustments to the parameters and keeps them only when they lead to a lower residual error value. In particular, it robustness is required for processing of quantized and disturbed measured values.

This trial-and-error search approach with many steps may not be efficient in terms of computational effort, but it is fully sufficient to achieve near real-time performance. Modern processors can still complete the localization calculations in few milliseconds, which is more than sufficient for the reaction time in the monitoring use case [21–23]. The stochastic optimization method is easy to implement in software. Additionally, it is suitable for future conversion to resource-constrained processors or microcontrollers.

**Fig. 9:**
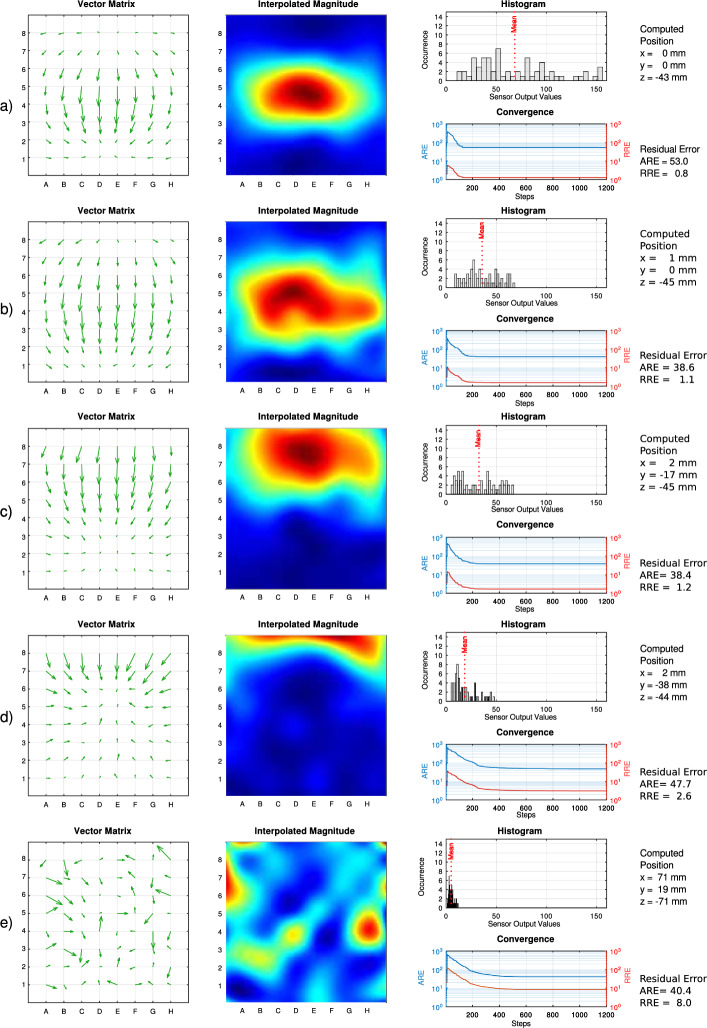
Visualization examples: **a**) Ideal scenario with the magnet positioned centrally. The histogram indicates excellent signal strength and well-balanced data distribution, leading to rapid and stable convergence toward a absolute residual error (ARE) and minimal relative residual error (RRE) that aligns with high signal strength. This setup is highly suitable as an initial reference, ensuring stable and accurate localization. **b)** Same as before, but less signal strength due to more distance from reference point. **c)** The magnet is shifted outward. The histogram shows usable signal strength and data distribution, with stable convergence. This position is less optimal as an initial reference, although it supports localization. **d)** The magnet is shifted far outward. This configuration is unsuitable as an initial reference and has less precise but possible localization. **e)** Scenario where the magnet is outside the detection range. The histogram shows insufficient signal strength and narrow data distribution and requires an alarm for this reason.

Robust convergence is maintained across varying magnetic source distances, including initial phases of ETT displacement dislocation, see also Fig.9.

### Extracorporeal fixation

The monitoring device with the sensor array must be attached external to the patient. To consider the limited detection range, the sensor array should be positioned as close as possible to the magnet. It is also necessary to ensure that the body surface provides stable support.

Furthermore, the influence of natural movements must be considered when designing the fixation. This is particularly relevant in the context of respiration, head position and patient transfer. The optimal placement of the ring magnet is in the vicinity of the cuff or slightly above it. This configuration allows for a short anatomical distance to the magnetic sensor array, which is advantageous.

Four suitable options are presented for placing the monitoring device extracorporeally at the patient:

Option 1: The fixation is frontally between the clavicles and above the distal end of the sternum. In individuals with a normal body shape, the monitoring device can be placed on a flat area of the body surface. However, this position has the disadvantage of being influenced by the natural breathing motion of the chest. The movement of raising and lowering the sensor array at this placement results in a cyclical alteration of its relative position with respect to the magnet.

Option 2: The frontal region of the neck is fitted with the device containing the planar sensor array. The short distance between the anterior neck surface and the magnet allows for minimal spacing, resulting in optimal signal strength. This is particularly relevant for very small ring magnets integrated into endotracheal tubes with small diameters. Compared to option 1, respiratory motion in this area is relatively limited. However, the available support surface for sensor placement is reduced. Since the monitoring unit with the planar sensor array is positioned on a curved anatomical surface, it must be properly supported and secured against tilting.

**Fig. 10:**
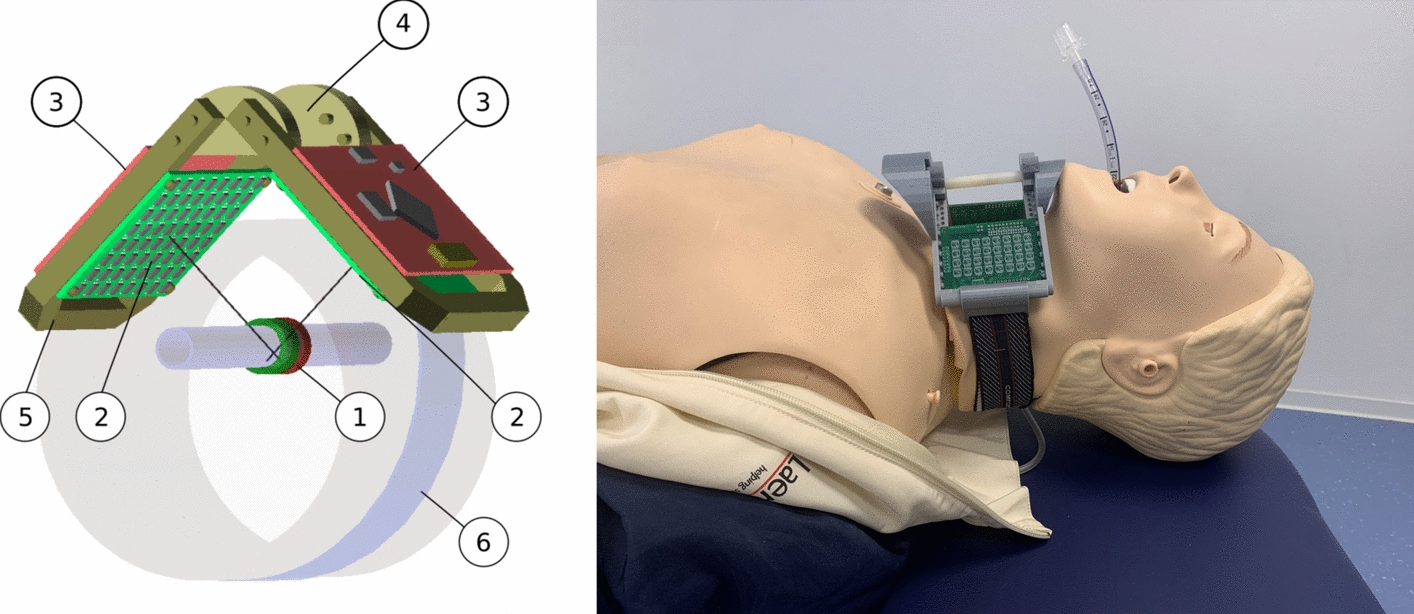
Proposal for fixation with a split array in angular position (Option 4). Left: Components arrangement: (1) Ring-magnet on the tube, (2) Circuit boards with right and left sensor array, (3) Circuit boards with microcontroller, (4) Central connecting elements (5) Two U-shaped plastic frames, (6) Flexible tensioning strap. Right: 3D-printed model placed on a phantom dummy

Option 3: A placement position as previously described in option 2, but the device is supported by a modified cervical immobilizer, also referred to as stiff neck. This modification allows stable attachment, preventing some of the natural movement. A front position of the sensor-array can be achieved with minimal modifications to the construction of the immobilizer. However, any additional distance between the magnet and the sensor-array may be disadvantageous. From a clinical perspective, the routine use of immobilizers in an ICU setting appears less feasible unless it is already intended for use in therapy.

Option 4: A fixation at the throat holds a split sensor array in an angled position. This configuration, which is formed of two sub-arrays, offers a number of advantages. It partially encloses the throat area and can be attached more stably with an elastic retaining strap. The arrangement of the sub-arrays allows a favorable small distance to the ring magnet from both sides. For this option, an illustration of a construction proposal and a model is provided in Fig. 10.

Initially we consider the option of an thin and bending flexible Printed Circuit Board (PCB) for the sensor array, which could offer advantages in terms of conformal placement.

However, we deliberately chose a rigid geometry in our sensor array. The signal processing and localization algorithm rely on the fixed knowledge of the spatial position of each sensor element. A flexible shape for the PCB entails the uncertainty of these positions.

### Visualization

All field visualizations presented in this work are based on experimental data acquired using the sensor array. The measured magnetic field values were processed and interpolated using in-house software implemented in GNU Octave and C to illustrate spatial magnetic field distributions.

For this study, two user interfaces were developed: one for the image similarity procedure and the other for the localization procedure. The software running on a personal or laptop computer presents proposals for the graphical visualization of the results.

For the image similarity method, the current measurement data are displayed as an interpolated image using a high-contrast color pallet. Each color represents the one magnitude value for the magnetic field data. The significant portion of the measured values with strong field strength is highlighted as a fuzzy indistinct cloud, which is highlighted in red. Medium magnitudes are shown in green and yellow. Low signal field strengths are shown in blue. The position of the highlighted cloud corresponds to the magnet position. Any movement is recognizable in real time, an example is illustrated in Fig. 6.

For the localization procedure, a spatial representation must be created. A three-dimensional projection is difficult to interpret. Therefore, the position is displayed in the three planes of the Cartesian coordinate system, for which three diagrams are required. In each of the three diagrams, the position is plotted as a point. This corresponds to the appropriate viewpoint on the plane. Additionally, it is beneficial to observe the magnet’s movement over an extended period and to plot it. The track of the plotted points corresponds to the trajectory of the movement. One option is to draw warning and alarm distance thresholds as circles or ellipses. This is shown in Fig. 7.

The convergence curve as the gradual reduction of the cost function can also be displayed. This is valuable for understanding the mode of operation or for adjusting the parameters of the search procedure. The value of the cost function at the end of the search should be relatively small compared to the average sensor signal strength. Otherwise, the localization procedure is considered unsuccessful and the result is invalid. This situation particularly arises when the magnet has moved beyond the effective sensing range.

**Fig. 11:**
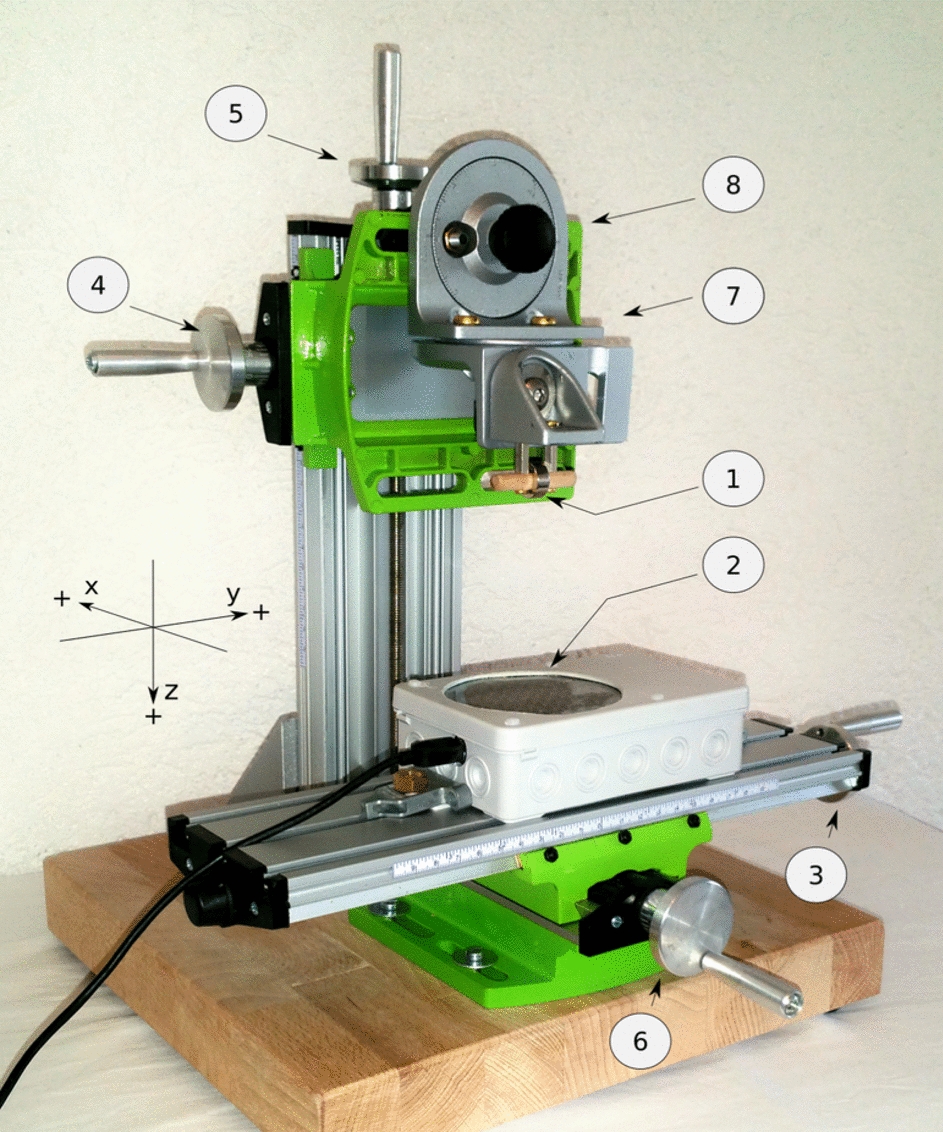
Measurement setup for simulated dislocation movement. (1) Ring-magnet, (2) Sensor array within device housing, (3) and (4) Adjusting wheel of Y-axis, (5) Adjusting wheel of Z-axis (6) Adjusting wheel of X-axis, (7) and (8) Tilt control

In addition to displaying the localization procedure convergence curve, the measured values can also be represented as a histogram.

This histogram reveals whether the raw data possesses sufficient resolution and distinct variations between values. Both the histogram and the relative residual error of the convergence curve, enabling users to evaluate the quality of measurement data and reliable localization. Examples can be found in Fig. 9.

This evaluation is crucial for ensuring the reliability of algorithms used for image comparison and localization. This assessment is essential when applying the fixation to the patient, as the initial reference image or position is recorded at this stage. It is vital that the reference information is accompanied by accurate and well-controlled measurement data, as it serves as the basis for all subsequent comparisons.

In events where an early warning or alarm is triggered, the graphic visualization can display additional details, such as the trajectory of the tube movement and the quality of the measurement data. These insights can assist in determining whether the tube is dislocated or if there has been a loss of fixation and a shift of the sensor array. Additionally, this visualization can aid in the system development and training of users interacting with the system.

### Test and evaluation

The image similarity method and the localization algorithm were tested with a series of experimental measurements. To evaluate the precision and repeatability, a series of measurements were taken in 10 mm increments along the three axes.

For the tube monitoring application, it is proposed to define this tolerance at ± 5 mm or less. This ensures that the usual accuracy of one centimeter for placement of the ETT in the references is maintained [1, 15, 32]. Measurements with the magnet of 5.6 g mass show that this applies up to a minimum Euclidean distance of 70 mm euclidean distance, with slightly wider tolerances of up to 90 mm. These distances are regularly possible at the preferred fixation position, as discussed before, which leads to reliable localization. Additionally, a small ring-shaped magnet with a mass of 2 g was experimentally characterized. It is suitable for tubes with a small diameter and achieves a localization detection range of 60 mm, which is sufficient when appropriately positioned.

A mechanical setup was constructed to enable the precise reproduction of positions and angular orientations between the sensor array and magnets. It uses two cross slides consisting of aluminum and without any ferromagnetic parts. The geometrical positions are mechanically set with a tolerance of ± 1 mm. Fig. 11 shows the configuration, which allows adjustment in all axes and tilt angles.

**Fig. 12:**
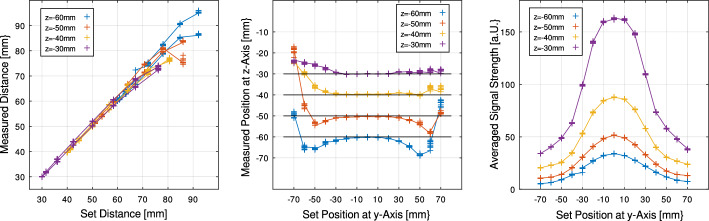
Example results: The measured values of a test series using the ring magnet with a mass of 5.6 g Neodym N52. Plots show results at prior stepwise presets at y-axis on the measurement setup. Left: Euclidean distances from magnet position Center: Position measured in different positions at z-axis Right: Average signal strength

This setup was employed in an experimental series investigating image processing and localization techniques. It also serves as a platform for testing the functionality of data processing algorithms. Fig. 12 illustrates few example results of the localization tests with mechanical setup.

Overall, the findings suggest that the technical parameters of the proposed system are sufficient to meet the task demands of the intended application.

## Data Availability

Dedicated data formats were used for the measurement series; the raw data are available from the authors upon request only.

## Abbreviations

Cartesian Coordinates: Defined by orthogonal X, Y, Z axes as three dimensions
Convergence Curve: Graph of residual cost function reduction over successive iterations of the localisation procedure
CT: Computer Tomography
DC Field: Directed current and continuously static magnetic field, also with permanent magnet source
ETT: Endotracheal Tube
Euclidean Distance: Straight-line distance between two points in two- or three-dimensional spaces
ICU: Intensive Care Unit
Inverse Problem: Reconstruction of unknown model parameters that caused observed measurement data; opposite of an forward problem, which computes measured data from known parameters
MRI: Magnetic Resonance Imaging
N40, N52: Material classification for permanent magnets of neodymium iron boron alloy
PCB: Printed Circuit Board
TMR: Tunnel Magnetoresistance: quantum effect where electrical resistance depends magnetic alignment across a thin barrier
X-Ray: Ionizing radiation imaging involving patient exposure
